# Development and Testing of an Adjuvant Radiotherapy Decision Aid for Older Women Diagnosed with Stage I Breast Cancer: A Pilot Study

**DOI:** 10.7759/cureus.7690

**Published:** 2020-04-16

**Authors:** Matt Neve, Nayanee Henry-Noel, Rajin Mehta, Maureen Trudeau, Ines Menjak, Ewa Szumacher

**Affiliations:** 1 Radiation Oncology, Sunnybrook Health Sciences Centre, Toronto, CAN; 2 Neuroscience and Health Studies, Population Health (Science), University of Toronto, Toronto, CAN; 3 Geriatrics, Sunnybrook Health Sciences Centre, Toronto, CAN; 4 Medical Oncology, Sunnybrook Health Sciences Centre, Toronto, CAN; 5 Radiation Oncology, University of Toronto, Toronto, CAN

**Keywords:** breast cancer, older women, radiation, radiotherapy

## Abstract

Background

Whole breast irradiation therapy (WBRT), accelerated partial breast irradiation (APBI), and omission of radiotherapy (ORT) are options for women aged 65 years and older with low-risk breast cancer post lumpectomy.

Aim

The aim of the study was to develop and pilot a decision aid pamphlet (DA), among women aged 65 years and older with low-risk breast cancer and who were undergoing or had undergone WBRT, to ensure they were fully informed about the different options for radiation treatment following lumpectomy.

Methods

We piloted the decision aid with 40 participants, women aged 65-86 years with low-risk breast cancer and who had undergone or were undergoing WBRT. The women completed a pre-DA Decisional Conflict Scale (DCS) and post-DA DCS, Knowledge, Preparation for Decision-Making and Acceptability questionnaires. We then used descriptive statistics to compare the DCS scores before and after distributing the decision aid.

Results

The median age of the 40 participants was 72 years (range, 65-86 years), 38% less than 70, 48% between 70 and 80 and 15% over 80. Ethnicity included 53% Caucasians and the remaining 48% African-Americans, Asians, Europeans, and others. Thirty-three percent completed high school, 25% college/university, and 7.5% elementary education. Seventy-eight percent had T1 and 23% T2 breast cancer. Thirty-three percent completed RT less than one year prior to the study, 30% between one to two years, and 38% greater than two years. The median pre-DA DCS score was 31.2 (31.2-90.6), and the median post-DA DCS score was 23.4 (0-75.0). Six (6/40) patients scored 0 on the DCS post intervention, while 13 (13/40) scored less than 15.6. The median knowledge score was 70%. Preparation for decision-making median score was 90%. Ninety-nine percent stated that the DA was useful for future patients.

Conclusion

We piloted a DA that aimed to provide the necessary information for women aged 65 years and older with low-risk breast cancer, to understand radiation treatment options post lumpectomy. The results obtained from the study highlighted the utility of the DA in increasing patient comprehension about the different treatment options, reducing decisional conflict in terms of perceptions of uncertainty and preparing patients to engage with their radiation oncologist during the treatment decision-making process. Ultimately, this study promoted the importance of patient-centered care in geriatric oncology by piloting this DA to see its effectiveness while also being responsive to patient’s thoughts regarding the tool, so as to have their values guide its further development.

## Introduction

Four out of 10 all new breast cancer cases are diagnosed in women aged 65 years and older, approximating 44.1% of new cases [1]. Furthermore, women in this age group are more likely to have biologically indolent cancers than those of the younger breast cancer patient population.

The standard management of care for women with early-stage breast cancer is lumpectomy. Historically, adjuvant whole breast radiation therapy (WBRT) was routinely administered based on gains in local control and overall survival rate, both indicated by the Early Breast Cancer Trialists’ Collaborative Group (EBCTCG) meta-analyses [2]. However, women aged 65 years and older were not well represented in these trials. As such, the outcomes of these trials among younger women had been extrapolated to older women with breast cancer.

It should be noted that the majority of recurrences in such patients are often found in the proximity of the original tumour - the lumpectomy cavity. In theory, the local control rates should be higher when receiving WBRT versus not receiving adjuvant radiation treatment. However, more recently, there is a high level of evidence suggesting that in the population of older women with stage 1 breast cancer, ER/PR positive and HER2 negative, the gains in ipsilateral local control are small, while other parameters, such as contralateral breast development, regional failures, and overall survival, are unchanged by the omission of adjuvant WBRT [3].

The PRIME I and PRIME II multicentre randomized controlled trials demonstrated that women aged 65 years and older, with early-stage breast cancer (axillary node-negative, T0-2, N0-1, M0 for PRIME I and ER expressed, axillary node-negative, T1-T2 for PRIME II) who have undergone lumpectomy, have low recurrence rates, as long as they are compliant with five years of anti-estrogen therapy [3-4]. These studies randomized participants to receive the standard adjuvant WBRT or to receive no radiation treatment. Follow up after five years in the PRIME I study indicated no difference in overall quality of life results between patients treated with WBRT and those who did not receive WBRT [4]. Similarly, the PRIME II study demonstrated no difference in overall survival and no difference in regional failure, distant metastases or contralateral breast development [3]. Furthermore, while there were improvements in ipsilateral breast cancer control rates with adjuvant WBRT, this absolute benefit was small: 1.3% ipsilateral breast tumour recurrence in the WBRT group versus 4.1% ipsilateral breast tumour recurrence in the omitted radiation treatment group [3].

The Cancer and Leukemia Group B (CALGB 9343) trial provided further evidence that radiation treatment can be safely omitted among patients aged 70 years and older with clinical stage I (T1N0M0) ER-positive breast cancers [5]. In this study, the absolute benefit was small with a 3% local control gain with WBRT noted at five years [5]. Similarly, the British Association of Surgical Oncology (BASO) II trial identified the risk of local recurrence to be 2.2% per annum for those who received surgery alone compared to a 0.8 % risk of local recurrence for those who received adjuvant radiotherapy [6].

In light of this evidence, the National Comprehensive Cancer Network (NCCN) guidelines list the omission of WBRT as a valid and safe option for women aged 65 years and older receiving adjuvant endocrine therapy [7]. 

Considering this evidence in older women, the concept of accelerated partial breast irradiation (APBI) may be appealing for such patients who are uncomfortable with the idea of not receiving adjuvant treatment. The smaller volume of tissue being irradiated should be associated with reduced toxicity, theoretically maximizing the therapeutic index given the low rates of local control benefit in this age cohort.

In fact, multiple studies including the GEC-ESTRO trial, University of Florence trial and the German-Austrian Multi-institutional Study have demonstrated low local recurrence rates (3.9% and 2.9%, respectively ) with APBI [8-10]. Furthermore, APBI provides the greatest reduction in radiation dosage to surrounding healthy tissues and reduces the duration of treatment from 3-7 weeks to approximately 2-5 days [8]. Overall, APBI has been shown to be as effective as whole breast irradiation with respect to controlling local recurrence amongst certain patients [11]. Based on the evidence, the American Society for Radiation Oncology (ASTRO) has published its support to the use of APBI in patients aged 50 years and older with low-risk breast cancer [11].

Taking this information into account, we decided it would be beneficial to include APBI in our decision aid (DA), despite it presently not being a standardized treatment of care. In recognizing that APBI may be a treatment option for future patients, as seen by ASTRO’s support, we believed it was important to include it so that we could examine the utility of the DA in increasing patient comprehension regarding this potential future treatment modality.

Currently, there are three potential adjuvant treatment options available for patients aged 65 years and older with low-risk breast cancer following lumpectomy. These include WBRT, accelerated partial breast irradiation (APBI), and omission of radiotherapy (ORT), provided the patient is compliant with adjuvant endocrine therapy.

Thus, the purpose of this study was to develop and pilot DA, among women aged 65 years and older with stage 1 breast cancer and who had undergone or were undergoing WBRT, to examine its utility as a resource in increasing patient knowledge, lowering decisional conflict and increasing patient preparedness such that they are fully informed about the different options for radiotherapy following lumpectomy.

## Materials and methods

Study population

We recruited all 40 participants from the clinic of a breast radiation oncologist at Sunnybrook Health Sciences Centre; all participants who were approached to participate agreed to take part in the study and met the eligibility criteria (Table 1).

**Table 1:** Study population

Age	Breast Cancer	Stage	Breast Cancer Characteristics	English Competency	Exclusion Criteria
Women 65 years and older	Infiltrating ductal carcinoma (IDC) Unifocal/unilateral distribution	G1-2; G3 (If LVI –ve); LVI +ve (If G3 is absent)	Estrogen receptor-positive (ER+), Her2-ve, all patients should be eligible for hormonal therapy with anti-estrogens	Must be able to speak/read English at a grade 8 level	Signs of mild cognitive impairment, signs of dementia, inability to communicate in English

Patients were eligible to participate if they were 65 years and older with stage I breast cancer and were previously treated with WBRT or were undergoing WBRT. We defined low-risk stage I breast cancer patients using the PRIME II study’s criteria: this included unifocal/unilateral infiltrating ductal carcinoma (IDC), grade 1 or 2 (if lymphovascular invasion LVI was present), grade 3 (LVI was not present), estrogen receptor (ER) positive, HER-2 negative. All participants were also required to be eligible for anti-estrogen medication. In addition to those requirements, participants also had to be able to speak and read English at a grade 8 level - those with cognitive impairment or who were unable to communicate in English were excluded from the study.

Testing of the DA among previously treated patients: There were multiple reasons as to why the DA was tested among previously treated patients including:

1. The aim of the study was to examine whether the DA was capable of increasing patient knowledge about the various treatment options so that it could be safely used among future patients during their treatment decision-making process.

2. The patient’s experience of having undergone or undergoing WBRT could provide valuable observations that could be added to the DA.

3. Access to patients; as the study was conducted within 12 months as a fellowship research project, we focused our accrual of patients from the follow-up breast cancer clinic.

4. In order to avoid conflict with the patient's treatment team and their decision-making process, we approach the patients after they discussed their treatment with their radiation oncologists.

5. At the time of this study, APBI was not a standard treatment option. As such, it was difficult and unethical to propose this method of treatment to patients who were currently in the treatment decision-making process.

Phase 1: Patient Decision Aid Development

We designed the trial following a thorough literature review along with feedback from a multi-disciplinary group of clinicians and allied healthcare providers involved in treating patients with breast cancer at the Sunnybrook Health Sciences Centre, in Toronto, Ontario, Canada. We developed the prototype of the decision aid (DA) and tested it using the Conceptual Framework of Decision Aid development guide from the Ottawa Hospital Research Institute. Subsequently, we employed evaluative tools designed and validated by the Ottawa Hospital Research Institute to produce quantitative and qualitative feedback regarding the utility of the DA tool. 

We received funding for the development of the DA from the Patient and Family Education Grant at Sunnybrook Health Sciences Centre.

Phase 2: Pilot Testing

We piloted the DA with a sample of 15 breast cancer patients. Appropriate changes were made based on patients’ feedback before we finalized the DA to use for the larger study. 

Phase 3: Field Testing

We tested the DA with 40 breast cancer patients and assessed the aid using all evaluative measures, designed and validated by the Ottawa Hospital Research Institute. The process of that testing is as follows:

1. After patients signed the Research Ethics Board (REB) consent form, the research assistant (RA) explained the concepts of WBRT, APBI and ORT. The RA followed a script to ensure that each patient was introduced to the study and the concepts in a standardized manner and therefore to assure a standardized, baseline knowledge.

2. The RA provided participants with a pre-DA ‘Decisional Conflict Scale’ (DCS) questionnaire that they answered using prior knowledge and information provided via the script. This was to formulate a baseline measure to assess personal perceptions of uncertainty in deciding choices, adjustable factors conductive to uncertainty and effective decision-making [12]. The DCS consisted of 16 items with five response categories from Strongly Agree to Strongly Disagree.

3. Post completion of the pre-DA DCS, the RA provided participants with the DA alongside the Evaluation Tools - post-DA DCS, Knowledge, Preparation for Decision Making Scale and Acceptability questionnaires.

4. The RA asked all participants to mail the questionnaires within four weeks of their initial meeting.

Structure of the Patient Decision Aid 

The DA was divided into three sections (Appendix 1):

1. Section One - How is low-risk breast cancer treated in women aged 65 years and older?

This section introduces the concept of low-risk breast cancer in patients 65 years and older. 

2. Section Two - What are my choices for radiation treatment after a lumpectomy? How can I decide?

This part outlines the radiation treatment options available at the Sunnybrook Health Sciences Centre, with diagrams illustrating the radiotherapy techniques of WBRT and APBI.

3. Section Three - Think about the benefits and risks

This section explains the best estimate of what happens to patients 65 years and over with low-risk breast cancer if they have either WBRT or no RT. In addition, there are pictures demonstrating the risks of local recurrence associated with each choice. It also outlines the benefits and risks/side effects of the various radiation treatment options. 

The DA was designed using the Ottawa Health Research Institute platform and is only available in English and aimed at a Grade 8 educational level. We consulted a literacy expert to ensure the absence of bias against lower educational levels and reduced English competency. Furthermore, we constructed the DA to comply with the International Patient Decision Aid Standards Checklist.

Evaluation of Tool Utility

To test the DA, we used the following evaluation tools from “The Ottawa Hospital Patient Decision Aids”

1. DCS: measures perceptions of uncertainty in deciding choices, adjustable factors contributing to uncertainty and effective decision-making. Scores range from zero (no decisional conflict) to 100 (extremely high decisional conflict) [12].

2. Knowledge questionnaire: focuses on information seen as necessary for decision-making. Specifically, it measures participant’s comprehension of a clinical issue, its rationale, benefits, risks and side effects alongside its substitutes. The result is given in a percentage value (%) [12].

3. Preparation for decision-making scale: measures participant’s perception of how effective a DA is in preparing them to engage with their physician regarding a health/treatment decision [12]. A higher score indicates a high-perceived level of preparation for decision-making, with a maximum score of 100% [12].

4. Acceptability questionnaire: focuses on ratings with respect to the comprehensibility of elements of a decision aid; the amount of information, balance in the presentation of information, length and the general effectiveness and suitability of the aid for decision making [12].

Statistical Analysis

We used descriptive statistics to analyse the results of the patient-reported assessments and we compared the DCS scores pre- and post-DA utilization. We consulted with the study biostatistician to conclude that no formal sample size calculations were required given that it was a descriptive pilot study.

## Results

Patient demographics

Age

Patients’ ages ranged from 65 to 86 years (median age = 71.5). Forty-eight percent of participants were aged between 70 and 80 years, with only 6 (15%) patients over the age of 80 (Table 2).

**Table 2:** Patient demographics

Age	
Median (range), years	71.5 (65-86)
<70	15
70-80	19
≥80	6
Ethnicity	Number of Patients
African-American	3
Asian	3
Caucasian	21
European	9
Education	Number of Patients
Completed Elementary School	3
Some High School	4
Completed High School	13
Some College/University	5
Completed College/University	10
Tumour Characteristics	Number of Patients
T1	31
T2	9
Grade 1	12
Grade 2	24
Grade 3	4
Treatment: Years Since RT	Number of Patients
<1	13
1-2	12
>2	15

Ethnicity

As expected, based on Toronto’s demographics, over half the respondents (53%) identified themselves as being of Caucasian ethnicity, while other ethnicities were more evenly represented (approximately 10% of respondents for each group - African American (7.5%), Asian (7.5%), European (23%), and Others 10%).

Education

The majority of respondents had either completed high school (33%) or college/university (25%) with a minority of respondents having solely completed elementary school (7.5%).

Tumour Characteristics

Of the participants recruited into the study, 31 (78%) had T1 and 9 (23%) had T2 breast cancer - all patients were node-negative.

Treatment

In terms of the years since radiotherapy among recruited patients, 13 (33%) completed their adjuvant radiotherapy less than one year prior to the study, 12 (30%) between one to two years before the study and 15 (38%) two or more years prior to the study.

Ottawa Decision Aid Tool Results

As indicated in Table 3, the median pre-DA DCS score was 31.2 with a range of 31.2 to 90.6. In comparison, the post-DA DCS demonstrated a reduction in the median score (23.4) and range (0-75.0) not statistically significant. Of the 40 participants, six participants scored zero on the DCS after receiving the DA indicating that all conflict with making a treatment decision was resolved after reading the DA. In addition, 13 participants scored less than 15.6 on the DCS after reviewing the DA indicating a low conflict with making a treatment decision.

**Table 3:** Ottawa patient decision aid results DCS, decisional conflict scale; DA, decision aid

Evaluation Measure	Range	Median	Mean
Pre-DA DCS	31.2-90.6	31.2	33.6
Post-DA DCS	0-75.0	23.4	20.9
Knowledge	15.0-95.0%	70.0%	68.3%
Preparation for Decision Making	55.0-100%	90.0%	85.5%

The participants demonstrated a wide variety of understanding of adjuvant breast cancer treatment options (median = 70% correctly answered questions). Preparation for decision-making tool demonstrated favorable results with a median score of 90.0% among participants. The acceptability questionnaire indicated high rates of satisfaction among participants; specifically, 99.0% of participants stated that the DA was clear and of potential value for future patients.

## Discussion

The DA was developed and piloted with the intention of providing the patient with a “resource to comprehend their disease and therapy options, to make higher quality decisions regarding their breast cancer treatment while demonstrating a commitment to great patient-centered care” [12]. The results from this study are in line with prior research that indicates DAs increase patient’s knowledge of their condition and treatment options, decrease passivity and indecision during medical encounters and benefit patient experience [13-14].

With respect to the utility of the DA, we used the DCS to measure perceptions of uncertainty from a patient’s perspective during the treatment decision-making process [12]. The reduction in median DCS score from 31.2 to 23.4 after participants reviewed the DA indicates that the tool was capable of providing valuable information and clarity regarding the different adjuvant radiation treatment options - WBRT, ABPI, and no RT. Further, the reduction in score indicates that patients perceived they were making an informed choice that was compatible with their personal values [12]. It also demonstrates that they perceived they were well supported and ultimately satisfied with their decision [12]. This conclusion is supported by Sun et al. (2005), who associated every DCS unit increase with a significant delay in making a decision and later changing the initial decision [15]. Additionally, poorer understanding and decisional regret were found to be associated with every DCS unit increase [15]. It is further supported by Gattellari and Ward (2004), who had identified a 19% greater probability of patients blaming their doctor for adverse outcomes for each unit increase in DCS and was an independent predictor of blame [16].

The Preparation for Decision-Making Scale evaluated patients’ perceptions of how effective the DA was in preparing them to engage with their radiation oncologist regarding treatment decisions [12]. Based on the information provided in the DA, our patients perceived the tool as being highly useful in preparing them and future patients in the treatment decision-making process [12]. Our patients approved of the DA’s ability to prepare them to communicate with their physician because it helped on a number of factors including helping them think about the benefits and risks associated with each option and thereby aiding them in thinking about which benefits and risks are most important [12]. Overall, our patients approved of the DA’s ability to reduce stress and improve confidence in making a treatment decision.

The ‘Knowledge’ questionnaire, which assessed patients’ awareness and knowledge of adjuvant breast cancer treatment options, their rationale, and the benefits and risks, had a high median score of 70% (15.0% to 95.0%). This finding could be related to the fact that all our patients were already treated with RT and may have had their own opinions about RT delivery. However, participants' levels of knowledge regarding the different treatment options for RT following lumpectomy differed and the information provided to them during their initial treatment decision-making process also varied. Furthermore, it was evident during the accrual process that most participants were not aware of APBI or that ORT was a safe option for women aged 65 years and older receiving adjuvant endocrine therapy [7]. As the script that was read contained only a brief overview of APBI and ORT but not a complete explanation of what they entail, many participants requested information about APBI and the safety of ORT.

Thus, while participants' prior knowledge may have slightly positively impacted their knowledge questionnaire results, their lack of knowledge regarding APBI and ORT should have skewed results negatively, which did not occur. It can then be reasonably concluded that the high median score of 70% was the result of the information provided in the DA. Therefore, this result supported the utility of the DA as a resource to increase participant’s awareness and comprehension of adjuvant breast cancer treatment options, their rationale, and their benefits and risks.

Strengths

Previous studies on adjuvant breast cancer therapies have often limited recruitment of women aged 65 years and older; therefore, our study’s focus on this demographic provided valuable information regarding their comprehension of the various options for radiation treatment post lumpectomy.

In addition, this study showed the utility of the DA in increasing knowledge about the different treatment options, in reducing perceptions of uncertainty and in increasing patient preparedness to communicate with one's radiation oncologist. As these three factors are connected with one another, the DA’s ability to improve one resulted in the betterment of the other. With respect to personal perceptions of uncertainty in deciding options, there are a number of adjustable factors that contribute to it - one prominent factor being feeling uninformed, which is influenced by knowledge [12]. The reduction of the DCS scores post reading the DA highlighted the utility of the DA in being able to increase patient knowledge and comprehension. As such, the DA helped reduce patients’ feelings of being uninformed about the various treatment options, thereby lowering feelings of uncertainty in choosing an option and decreasing decisional conflict.

Other strengths of the study include the development of an ‘in house’ DA tool, utilizing local staff and literacy experts who made it representative of the local population. In addition, the study tested the DA on patients who had either completed or were receiving treatment to the whole breast as part of their adjuvant treatment. Thus, as these patients had experienced the challenges of making the treatment decision, they provided a reliable source of feedback and quantitative data.

Ultimately, our study importantly promotes the concept of patient-centered care in geriatric oncology. For one, by piloting this DA, we were able to identify its utility in providing the necessary and essential information to patients in order for them to actively participate, to the extent they would like, in the treatment decision-making process. Furthermore, during this process, we ensured that we were respectful and responsive to patient’s thoughts, preferences, and needs with regards to the DA, such that their values guided its further development [17].

Limitations

This study has several limitations: the retrospective nature and the subsequent presence of selection bias. Since our patient cohort had either received or was being treated with whole-breast irradiation, there was a bias towards them preferring the concept of APBI to no radiotherapy.

The DA was viable in its ability in providing information regarding the different treatment options; however, APBI was not a standardized treatment option and so it was unethical to pilot it among patients currently in the treatment decision-making process.

Patient comments regarding the DA during the accrual process

Participants had expressed concern over the fact that they had either completed treatment or were undergoing treatment and thus did not know whether to answer the pre-DA DCS questions as they were presently (post-treatment) or as they were before (pre-treatment). This was a valid concern and the research assistant informed the participants that they should place themselves in a theoretical position where they have not undergone treatment. Furthermore, participants were informed to maintain that mindset during the process of reading the DA and during the completion of the subsequent evaluative questionnaires. As this concern was brought up relatively early during the accrual process, we changed the oral script to ensure that participants were aware that they were to place themselves in the theoretical situation of pre-treatment/not having undergone treatment.

Empowering older women with breast cancer to participate in the treatment decision-making process is globally relevant considering the increasing aging population. However, there are challenges associated with treating this age group of patients related to a number of factors including health literacy, education, and social supports. Furthermore, the more passive participation among women aged 65 years and over with low-risk breast cancer during the treatment decision-making process could result in a ‘missed opportunity’ to comprehend the available treatment options and choose an option based on their preferences. There is also the risk of creating further stress and confusion when presenting patients with multiple treatment options.

Therefore, effective communication of medical information with patients is fundamentally important, as it has been demonstrated to impact a patient’s awareness of prognosis and their choice during the treatment decision-making process. Focusing on and utilizing the knowledge and personal perceptions of patients, we developed and piloted a DA that aimed to provide the necessary information for patients to better understand their disease and treatment options for adjuvant RT post lumpectomy and thereby allow them to make higher quality choices.

Future directions

Further large-scale trials are required to assist in assessing the validity of utilizing this DA before it can be recommended to be formally provided to women aged 65 years and over with early breast cancer. Pleasingly, we are aware that there is a large randomized controlled trial being undertaken at Dana Ferber, looking at an almost identical population [18].

## Conclusions

In conclusion, this study promotes the concepts of patient-centered care, education, and increased patient participation in treatment decision-making. Future studies to validate our DA are the next steps to further promote such valuable concepts.

## Figures and Tables

**Figure 1:**
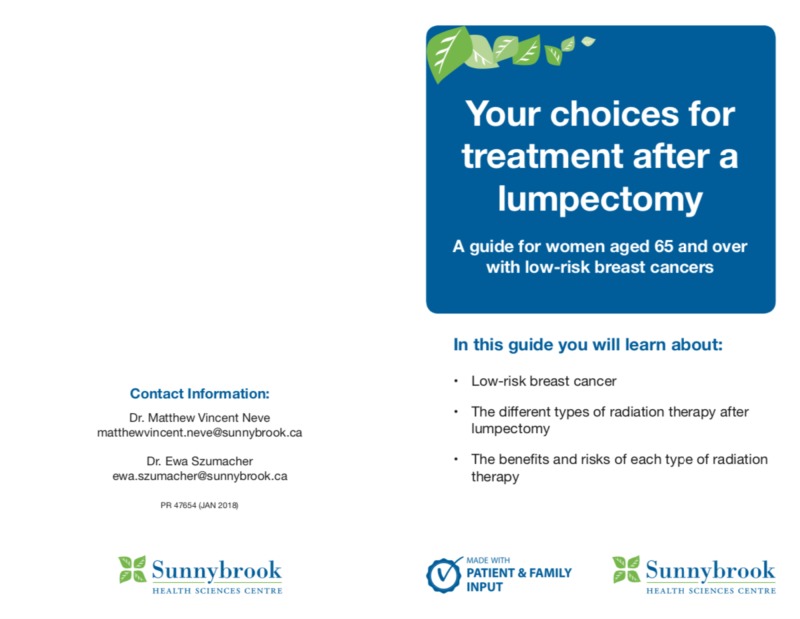
Decision aid (page 1 and page 8)

**Figure 2:**
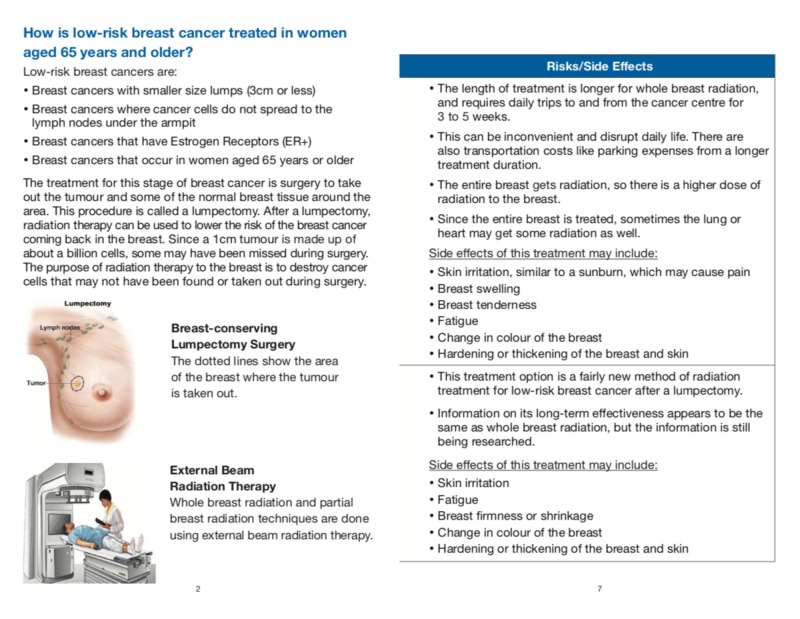
Decision aid (page 2 and page 7)

**Figure 3:**
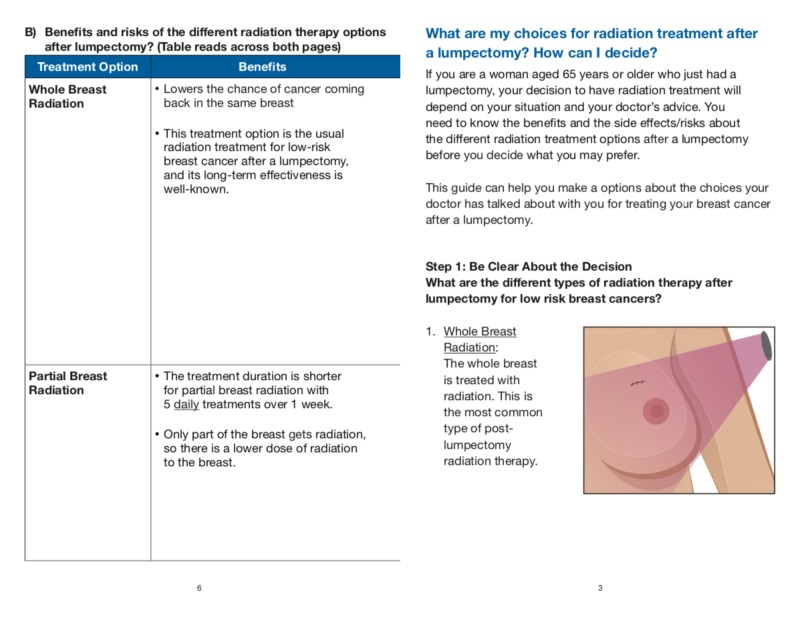
Decision aid (page 3 and page 6)

**Figure 4:**
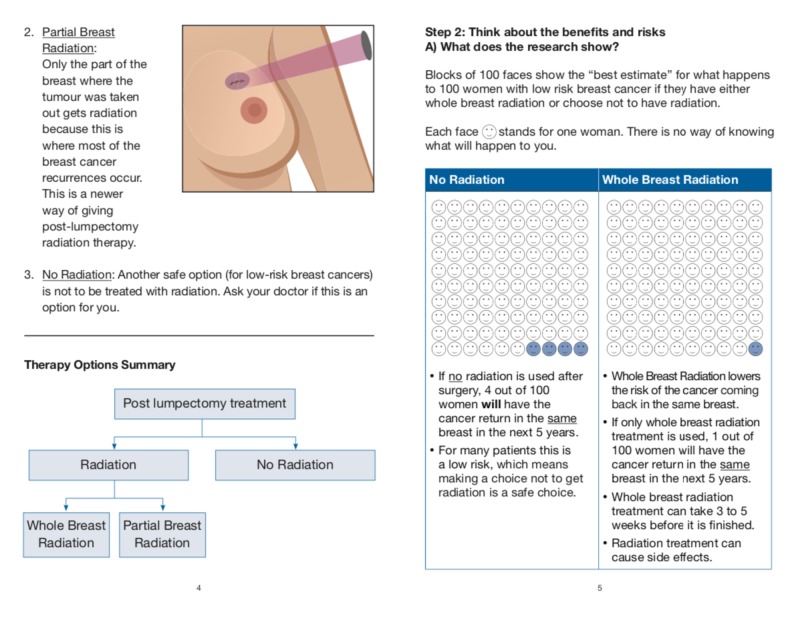
Decision aid (page 4 and page 5)
